# ﻿Phylogenetic and taxonomic studies of six recently-described *Stellaria* species (Caryophyllaceae) from China, with an additional new species, *Stellarialongipedicellata*, from Sichuan

**DOI:** 10.3897/phytokeys.249.136456

**Published:** 2024-12-05

**Authors:** Wenqiao Wang, Zhiwei Su, Zhonghui Ma

**Affiliations:** 1 College of Agriculture, Guangxi University, Nanning 530004, China Guangxi University Nanning China

**Keywords:** Alsineae, new combination, new species, *Stellaria*

## Abstract

The phylogenetic studies of the tribe Alsineae (Caryophyllaceae) have revealed a clearer boundary between the genus *Stellaria* and related genera, primarily relying on the morphological characteristics of style 3, stamens 10 and petals deeply bifid. However, the newly-published species in China, which have 5 styles or ten or more lobes per petal, challenge this boundary and necessitate further studies. In this paper, we reviewed six newly-published Chinese species of *Stellaria*, utilising both molecular phylogenetic evidence from nuclear ribosomal internal transcribed spacer (ITS) and four plastid regions (*trnL-F*, *matK*, *rbcL*, *rps16*) and morphological evidence. Our results demonstrated that the five new species (*Stellariaabaensis*, *S.multipartita*, *S.pentastyla*, *S.procumbens* and *S.zhuxiensis*) were nested within the genus *Stellaria*, but *Stellariamotuoensis* was sister to the genus *Schizotechium*. Herein, we accepted four new *Stellaria* species and proposed a new combination in *Schizotechium* and a new synonym in *Stellaria*. Additionally, we described a new species *Stellarialongipedicellata* from Sichuan Province, China, which was distinguished from the closely-related species *Stellariadecumbens* by its glabrous body, linear-lanceolate leaves, long pedicellate flowers, prostrate growth habit and flowers nearly equal to or slightly shorter than sepals. Both molecular and morphological evidence support the treatment of *S.longipedicellata* as a new species of the genus *Stellaria*.

## ﻿Introduction

The classification of three subfamilies has been widely accepted in the family Caryophyllaceae (Lu et al. 2001). Since the establishment of phylogenetic tree of Caryophyllaceae, the classification of eleven tribes has been increasingly accepted (Harbaugh et al. 2010; Greenberg and Donoghue 2011). The tribe Alsineae, as a major branch of Caryophyllaceae with a great diversity of species, has attracted much attention. Recent studies on the tribe Alsineae have clarified the boundaries of its genera and have described the following new genera: *Engellaria* Iamonico, *Hartmaniella* M.L.Zhang & Rabeler, *Nubelaria* M.T.Sharples & E.A.Tripp, *Rabelera* M.T.Sharples & E.A.Tripp, *Shivparvatia* Pusalkar & D.K.Singh, *Hesperostellaria* Gang Yao, B. Xue & Z.Q. Song, *Reniostellaria* Gang Yao, B. Xue & Z.Q. Song and *Torreyostellaria* Gang Yao, B. Xue & Z.Q. Song (Zhang et al. 2017; Sharples and Tripp 2019; Iamonico 2021; Pusalkar and Singh 2022; Xue et al. 2023). *Stellaria* L. is particularly noteworthy as one of the genera with a high species richness in the tribe Alsineae, mainly characterised by 4 or 5 petals, petals usually deeply bifid (rarely retuse or multilobed) and capsules opening by valves 2 times number of styles (Chen and Rabeler 2001). Despite the species richness of this genus, limited research has been conducted on it. Previous phylogenetic studies of the family Caryophyllaceae have proposed that the traditional *Stellaria* was paraphyletic (Harbaugh et al. 2010; Greenberg and Donoghue 2011; Dillenberger and Kadereit 2014; Zhang et al. 2017). Due to the global studies on *Stellaria*, its classification has been clarified and species with non-deeply-bifid petals or 2 styles have been assigned as the following new genera: *Nubelaria*, *Rabelera*, *Hesperostellaria*, *Reniostellaria* and *Torreyostellaria* (Sharples and Tripp 2019; Xue et al. 2023). Then the core *Stellaria* in a new circumscription can be clearly distinguished from other related genera in having deeply-bifid petals (sometimes absent) usually in combination with six-valved capsules (Sharples and Tripp 2019). However, there is still a lack of research on new species of *Stellaria* with distinctive petals found in China.

In recent publications, six new *Stellaria* species have been documented in China, described as *Stellariaabaensis* H.F. Xu & Z.H. Ma, *S.motuoensis* Meng Li & Y.F. Song, *S.multipartita* Bo Xu & Meng Li, *S.pentastyla* W.Q. Wang, H.F. Xu & Z.H. Ma, *S.procumbens* Huan C. Wang & Feng Yang and *S.zhuxiensis* Q.L. Gan & X.W. Li. (Gan and Li 2014; Xu and Ma 2018; Song et al. 2020; Wang et al. 2020; Yang et al. 2020; Li et al. 2022). Amongst them, only three species (*S.motuoensis*, *S.multipartita* and *S.pentastyla*) were supported by the phylogenetic study, while the precise phylogenetic position of the remaining species requires further investigation. Notably, four species exhibit distinct flower morphology compared to the core *Stellaria* species. For instance, *S.motuoensis* lacks petals and bears 5 stamens, *S.multipartita* displays ten or more lobes per petal, while *S.pentastyla* and *S.procumbens* have 5 styles. Furthermore, two species share similar morphological characteristics with related species, such as *S.abaensis* and *S.petiolaris* Hand.-Mazz., *S.zhuxiensis* and *S.vestita* Kurz. Further in-depth research is crucial for a comprehensive understanding of these newly-named *Stellaria* species.

In addition, during a field survey in Sichuan Province of China, we discovered an undescribed species whose morphology does not correspond to any known *Stellaria* species. Based on detailed morphological and molecular studies, we hereby describe it as a new species.

## ﻿Materials and methods

### ﻿Sample and morphology

In this study, we sampled major genera of the tribe Alsineae and major clades of *Stellaria* in order to accurately determine the phylogenetic position of the new species, with *Arenariaserpyllifolia* L. serving as the outgroup. Samples and accession numbers are listed in Table 1, Suppl. material 1. The morphological traits of the new species were examined by either the original specimens or their images and also by specimens collected through our own field surveys.

**Table 1.** Taxa sampled and the vouchers.

Taxon	Location	Latitude, Longitude	Collector and number	Herbarium
*Stellariaabaensis* H.F. Xu & Z.H. Ma	Tianquan, Sichuan	31.049472, 102.874177	H.F. Xu SC0037	GAUA
*Stellariaamplexicaulis* (Hand.-Mazz.) Huan C. Wang & Feng Yang	Luding, Sichuan	26.016249, 98.620941	H.F. Xu & G.F. Mou YN0002	GAUA
*Stellariaprocumbens* Huan C. Wang & Feng Yang	Luding, Sichuan	29.851945, 102.286631	W.Q. Wang et al. QSC0007	GAUA
*Stellariaradians* L.	Yakeshi, Neimenggu	49.327739, 120.676409	W.Q. Wang & R. Wu NM0010	GAUA
*Stellarialongipedicellata* W.Q. Wang & Z.H. Ma	Luding, Sichuan	29.863690, 102.289755	W.Q. Wang et al. QSC0009	GAUA
*Stellariazhuxiensis* Q.L.Gan & X.W.Li	Zhuxi, Hubei	32.436378, 109.561435	W.Q. Wang & Z. Xie HB0034	GAUA

### ﻿Phylogenetic analysis

Total DNA was extracted from silica gel dried leaves by modified CTAB (Doyle and Doyle 1987). Subsequently, we performed PCR amplification of the following markers as cited, ITS (5F, 4R), *matK* (390F, 1440R), *rbcL* (1F, 724R), *rps16* (F, R) and *trnL-F* (C, F) (White et al. 1990; Taberlet et al. 1991; Popp and Oxelman 2001; Smissen et al. 2002; Kress and Erickson 2007). The PCR products were then sequenced by the Beijing Genomics Institute (BGI). The obtained sequences were double-stranded spliced and checked using GENEIOUS v.11.0.4 (Kearse et al. 2012) and the phylogenetic trees were conducted using PHYLOSUITE v.1.2.2 (Zhang et al. 2020). Initially, the sequences were aligned by MAFFT v.7.313 (Katoh and Standley 2013). Then we used PARTITIONFINDER v.2.1.1 (Lanfear et al. 2016) to determine the best model under the Akaike Information Criterion (AIC). The selected models were SYM+I+G for ITS, GTR+G for *matK*, *trnL-F*, and *rps16* and HKY+I+G for *rbcL*. The Bayesian Inference (BI) trees were constructed using MrBayes 3.2.6 (Ronquist et al. 2012) with 2,000,000 generations and the tree sampled every 100 generations. The first 25% trees of each run were discarded as burn-in. To assess the chain convergence, it was verified that the average standard deviation (SD) of the split frequencies was below 0.01. Finally, Maximum Likelihood (ML) trees were constructed using the GTRGAMMA model with 1,000 bootstrap replicates and default values for the remaining parameters on the CIPRES Science Gateway (Miller et al. 2010). No notable incongruence was found between the nrITS phylogenetic tree and the plastid phylogenetic tree.

## ﻿Results

**Figure 1.**
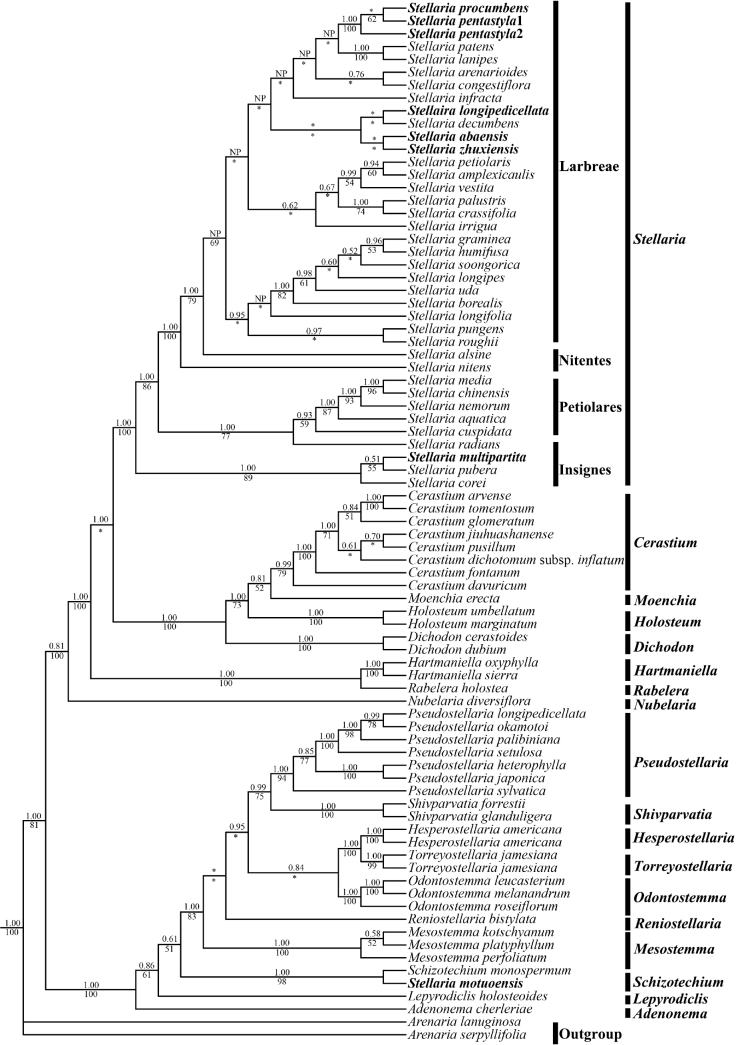
Maximum likelihood (ML) tree of Alsineae inferred from the Alsineae-wide dataset (including nrITS, *matK*, *rbcL*, *rps16* intron and *trnL-F* intergenic region). Posterior probability (PP) in Bayesian Inference (BI) and bootstrap (BS) value in ML analysis are indicated above and below the stem branch of each phylogenetic node, respectively. NP indicates the topology was not present in BI analysis. * indicates that the PP or BS value is less than 0.5 or 50%.

In the phylogenetic tree of the tribe Alsineae (Fig. 1), *Stellaria* formed a single clade, except for *Stellariamotuoensis*. Our results revealed that five new species (*S.abaensis*, *S.multipartita*, *S.pentastyla*, *S.procumbens* and *S.zhuxiensis*) were nested within *Stellaria*, but *S.motuoensis* was sister to *Schizotechium* with high support (BS = 100%, PP = 1.00). *Stellariaprocumbens* was nested within the Larbreae clade and identified as the sister to *S.pentastyla* (BS = 100%, PP = 1.00). Notably, *S.abaensis* and *S.zhuxiensis* fell into the Larbreae clade and were sister to each other. Then *S.multipartita* was clustered with *S.pubera* Michx. and *S.corei* Shinnersin, forming the *Insignes* clade. Finally, *S.longipedicellata*, a potential new species was sister to *S.decumbens* Edgew.

## ﻿Discussion

Sect. Schizothecium Fenzl, a traditional section of the genus Stellaria, is characteried by having 3 styles and 1–2 seeds. It includes *S.delavayi* Franch., *S.monosperma* Buch.-Ham. ex D. Don and *S.ovatifolia* (Mizushima) Mizushima (Wu and Ke 1996). However, recent studies proposed that sect. Schizothecium should be resurrected as the genus *Schizotechium* (Pusalkar and Srivastava 2016; Arabi et al. 2022; Wang et al. 2023; Xue et al. 2023). Consequently, *Stellariadelavayi* and *S.monosperma* of sect.Schizothecium were reclassified under the genus *Schizotechium* (Pusalkar and Srivastava 2016; Xue et al. 2023). Moreover, it has been suggested that *S.ovatifolia* should revert to its previous name *Brachystemmaovatifolium* M. Mizush. (Wang et al. 2023). When *S.motuoensis* was published, it exhibited morphological similarities with S.monospermavar.paniculata (Edgew.) Majumdar and was sister to *S.monosperma* in the phylogenetic tree (Li et al. 2022). However, authors still considered *S.motuoensis* as part of *Stellaria* due to uncertainty of the phylogenetic tree of the tribe Alsineae and its morphological differences with *Schizotechium* species (Li et al. 2022). Nonetheless, more recent studies on the tribe Alsineae have accepted the genus *Schizotechium* (Pusalkar and Srivastava 2016; Arabi et al. 2022; Wang et al. 2023; Xue et al. 2023). Our study also indicated that *S.motuoensis* was nested within *Schizotechium* and sister to *Schizotechiummonosperma* with high support (BS = 100, PP = 1.00, Fig. 1), which is consistent with previous studies (Li et al. 2022). Importantly, *S.motuoensis* (with many-flowered compounds cymes, 5 stamens, 2–3 styles and 1–3 seeds; see Table 2) is morphologically more similar to *Schizotechium* (with many-flowered compounds cymes, 5 stamens, 2–3 styles and 1–3 seeds compound cymes, 5 or 10 stamens, 2–3 styles and 1–6 seeds; see Table 2), rather than the core *Stellaria* (with lax dichasial cymes, rarely solitary, 10 stamens, 3 or 5 styles and numerous seeds) (Pusalkar and Srivastava 2016; Arabi et al. 2022, Li et al. 2022). Based on the morphological and phylogenetic evidence, we propose a new combination for *Stellariamotuoensis*.

**Table 2.** Morphological comparisons of six species.

	*S.motuoensis*	*Schizotechiummonosperma*	*S.procumbens*	*S.pentastyla*	*S.longipedicellata*	*S.decumbens*
Petal	absent	2-lobed to middle	deeply bifid	deeply bifid	deeply bifid	deeply bifid
Stem	glabrous basally, pubescent in apical part	pubescent with 1 or 2 rows of glandular hairs above	glabrous	glabrous	glabrous	densely white pubescent
Style	2–4	3	3 or 5	5	3, rarely 4	3
Leaf	shortly petiolate, lamina ovate or oblong	short or long petiolate, lanceolate or oblong-lanceolate to elliptic	sessile, linear or acicular	sessile, linear	sessile, linear-lanceolate	sessile, oblong
Seed	1–3	1–2	numerous	numerous	numerous	numerous
Pedicel	pubescent	pubescent	glabrous	glabrous	glabrous	pubescent
Stamen	5	10	10	10	10	10

Presence of the stellate hair is a highly distinctive character in *Stellaria* including *S.vestita*, *S.infracta* Maximowicz and *S.amplexicaulis* (Hand.-Mazz.) Huan C.Wang & Feng Yang (Chen and Rabeler 2001; Yang et al. 2020). *Stellariazhuxiensis* closely resembles *S.vestita* with its sessile leaf and dense stellate indumentum, but differs by its longer petals and ovate leaves (Gan and Li 2014). Interestingly, *S.zhuxiensis* was sister to *S.abaensis*, not to any other of the stellate-haired species (Fig. 1). Our findings were consistent with the previous studies, which indicated that *S.vestita* and *S.infracta* with stellate hairs were not sister taxa on the phylogenetic tree (Sharples and Tripp 2019). It could be attributed to the parallel evolution of stellate trichomes (Sharples 2019).

The classification of ser. Petiolares is widely accepted within *Stellaria*, characterised by the presence of distantly petiolate leaves (Endlicher 1840; Schischkin 1970; Wu and Ke 1996). The latest *Stellaria* phylogenetic study also kept the classification of the *Petiolares* clade (Sharples and Tripp 2019). *Stellariaabaensis* with long petioles was closely similar to the *Petiolares* clade species (Xu and Ma 2018). However, it is intriguing to discover that *S.abaensis* nested in the Larbreae clade instead of being associated with the *Petiolares* clade (Fig. 1). Similarly, being consistent with the previous studies (Sharples and Tripp 2019), petiolate species such as *S.vestita* and *S.petiolaris* were placed in the Larbreae clade rather than the *Petiolares* clade (Fig. 1). Hence, the presence of petiolate or sessile traits may not be reliable indicators for classification within *Stellaria*. Additionally, it is worth noting that *S.abaensis* was often misidentified as *S.petiolaris* before its official publication. For instance, *S.capillipes* (Franch.) C. Y. Wu (the synonymy of *S.petiolaris*) referred numerous specimens of *S.abaensis* and the description and pictures of *S.petiolaris* in the Flora of Yunnan actually corresponded to *S.abaensis* (Wu 1995). However, *S.abaensis* can be easily distinguished from *S.petiolaris* due to its glabrous leaves and long petiole with ciliate hair (vs. leaves and short petiole densely covered with white villous hair), glabrous plant body (vs. plant body densely covered with white villous hair), petal lobes ovate-oblong (vs. lobes narrowly linear) and the capsule longer than persistent sepals (vs. capsule ca. 1/2 as long as persistent sepals) (Chen and Rabeler 2001; Xu and Ma 2018).

The traditional *Stellaria* did not include species with five styles, which is commonly found in the related genus *Cerastium* L. (Chen and Rabeler 2001). However, since *S.aquatica* (L.) Scop. (*Myosotonaquaticum* (L.) Moench) was included within *Stellaria*, the genus *Stellaria* recently also includes five-styled species (Sharples and Tripp 2019; Wang et al. 2020; Xu et al. 2020; Yao et al. 2021; Arabi et al. 2022; Xue et al. 2023). This study revealed that *S.pentastyla* and *S.procumbens* bearing 5 styles are not sister to the clade including *S.aquatica* (*Petiolares* clade), but constitute a distinct clade (Fig. 1). This suggests the existence of a new group within *Stellaria*. Notably, *S.pentastyla* and *S.procumbens* share morphological similarities (refer to Table 2) such as linear leaves, glabrous stems and 5 styles (Wang et al. 2020; Yang et al. 2020). Moreover, they have overlapping distribution (Lushui, Yunnan) and similar altitudinal distribution range (2100–3800 m). After comparing the field and specimen morphology of *S.procumbens* and *S.pentastyla*, it was found that there was no significant difference between the two. It indicates that *S.procumbens* and *S.pentastyla* are not two distinct species, but the same species. Furthermore, our phylogenetic result supports this conclusion that *S.procumbens* and *S.pentastyla* form a strongly-supported clade (BS = 100, PP = 1.00, Fig. 1). Considering the publication date of *S.pentastyla* (4 March 2020) preceding that of *S.procumbens* (9 March 2020), we propose that *S.procumbens* should be treated as a new synonym of *S.pentastyla* according to the Art. 11. 4 of the Shenzhen Code (Turland et al. 2018).

Having five or more lobes per petal is an exceptional character state in *Stellaria*, with *Stellariaradians* L. as the only species exhibiting this trait (Chen and Rabeler 2001). Despite its unique floral morphology, *S.radians* is widely recognised as a member of *Stellaria* (Schischkin 1970; Wu and Ke 1996; Chen and Rabeler 2001). Previous phylogenetic studies also indicated that it was nested in core *Stellaria* and formed an *Insignes* clade with other *Stellaria* species (Sharples and Tripp 2019). Our results revealed that *S.multipartita* is another species of *Stellaria* which possesses ten or more lobes per petal because it formed a clade with *Stellariapubera* and *S.corei* (Fig. 1). The previous study indicated *S.radians* formed a clade with *S.pubera*, *S.corei* and *S.sessiliflora* Y.Yabe (Sharples and Tripp 2019). It implied the close relationship between *S.radians* and *S.multipartita*. While *S.multipartita* shares similar petals with *S.radians*, it differs significantly in terms of petal characteristics (10–12-cleft vs. 5–7-cleft in *S.radians*) and distribution (Chongqing vs. Hebei, Heilongjiang, Jilin, Liaoning and Neimenggu in *S.radians*). This difference, as well as our phylogenetic data, support the treatment of *S.multipartita* as a new *Stellaria* species.

### ﻿Taxonomic conclusions

#### ﻿ Stellaria pentastyla

Taxon classificationPlantaeCaryophyllalesCaryophyllaceae

W.Qiao Wang, H.F.Xu & Z.H.Ma, Phytotaxa 435: 71. [4 March] 2020. Type: CHINA. Yunnan: Lushui, elev. ca. 3102 m, 25°58'26"N, 98°40'44"E, 8 June 2017, Xu & Mou YN0014 (holotype GAUA!, isotypes IBSC!)

8373ADCC-758C-5527-9C01-232F714D20A4

= Stellariaprocumbens Huan C.Wang & Feng Yang, Phytotaxa 435: 195. [9 March] 2020. Type: CHINA. Yunnan Province: Luquan County, Zhuanlong Town, Jiaozishan National Nature Reserve, 26°04'58"N, 102°51'04"E, elev. 3380 m, 12 July 2019, H. C. Wang et al. LQ 7217 (holotype YUKU!, isotypes YUKU!), syn. nov.

#### ﻿ Schizotechium motuoensis

Taxon classificationPlantaeCaryophyllalesCaryophyllaceae

(Li & Song) W.Q.Wang & Z.H.Ma comb. nov.

31E69A5A-2CE4-5112-A801-38C09AAE9331

urn:lsid:ipni.org:names:77352937-1

≡ Stellariamotuoensis Meng Li & Y.F.Song, Nordic J. Bot. 2022(9)-e03683: 2. 2022. Type: China, Xizang, Motuo County, Mt Doxong La, Xiaoyandong, ca. 2650 m a.s.l., 95°04'10.33"E, 29°24'51.13"N, 22 October 2021, Meng Li 3021 (holotype: NF, isotypes: CDBI).

#### ﻿ Stellaria longipedicellata

Taxon classificationPlantaeCaryophyllalesCaryophyllaceae

W.Q.Wang & Z.H.Ma sp. nov.

022CB8EA-CF80-52BB-BFE5-9C3E76BE7F25

urn:lsid:ipni.org:names:77352938-1

Fig. 2

##### Type.

China • Sichuan: Luding, growing on scree slopes, elev. ca. 2881 m, 29°51'49.2"N, 102°17'23.0"E, 8 July 2022, *W.Q. Wang et al. QSC0009* (holotype GAUA!).

**Figure 2.**
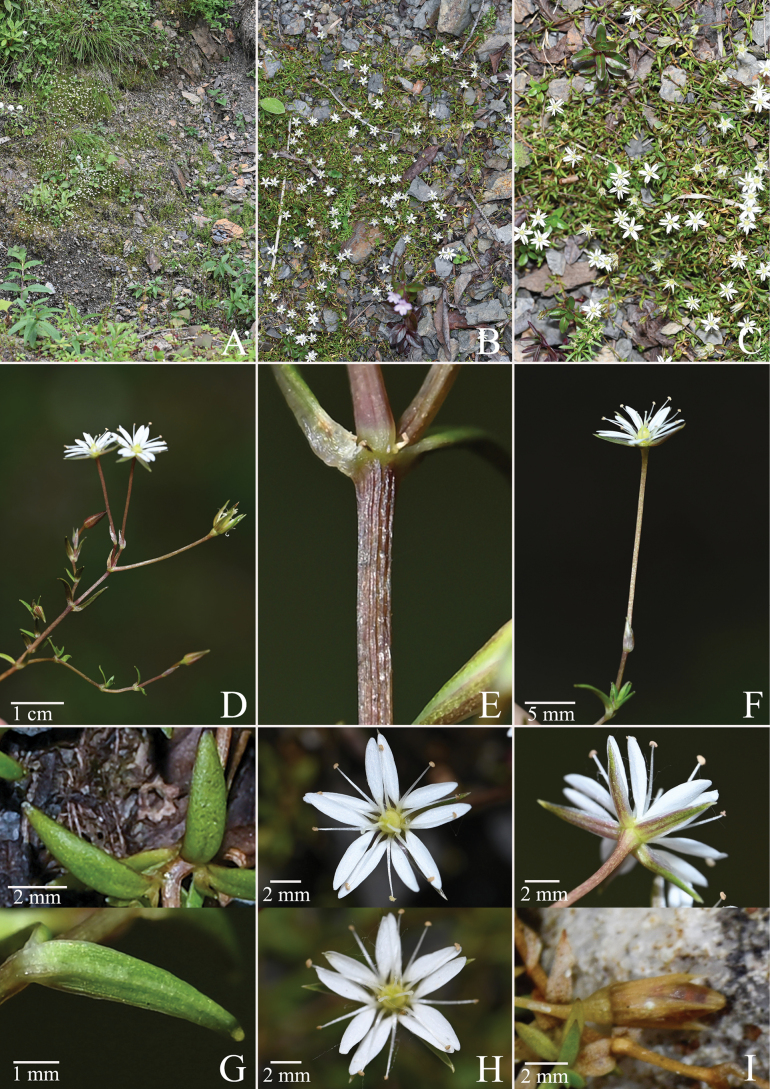
Morphology of *S.longipedicellata***A** habitat **B** habit **C** habit **D** inflorescence (cymes) **E** stem **F** inflorescence (solitary) **G** leaf **H** flower (style 3, rarely 4) **I** capsule.

##### Diagnosis.

*S.longipedicellata* is similar to *S.decumbens* in having a prostrate life form and few-flowered cymes or solitary flowers, but differs from the latter by having glabrous stems (vs. densely pilose stems), linear-lanceolate leaves (vs. oblong leaves), 1.2–2 cm pedicel, longer than sepals (vs. ca. 4 mm or less pedicel, shorter than or equalling sepals) and petals slightly shorter than or sub-equalling petals (vs. petals ca. 1/2 as long as sepals, in Table 2).

##### Description.

Perennial herbs, whole plants glabrous. Stems, slender, prostrate or slightly ascending, much branched, 5–10 cm tall. Leaves sessile, green, linear-lanceolate, minute, 3–7 mm long, 0.6–1 mm broad, apex acute. Inflorescence axillary or terminal, 1–3-flowered. Pedicel 1.2–2 cm, slender. Bracts lanceolate, 3–4 mm, margin membranous. Sepals 5, glabrous, lanceolate, 4–5 mm long, 0.8–1 mm broad, margin membranous, apex acuminate. Petals 5, slightly shorter than or subequalling sepals, deeply bifid. Stamens 10, slightly shorter than or subequalling petals. Styles 3, rarely 4, filiform. Capsule ovoid-cylindrical, slightly longer than or subequalling persistent sepals. Seeds numerous, red-brown, ovoid, conspicuously rugulose.

##### Phenology.

Flowering time June–July, fruiting time August–September.

##### Distribution and ecology.

It is only known from the type locality, growing on scree slopes.

## Supplementary Material

XML Treatment for Stellaria pentastyla

XML Treatment for Schizotechium motuoensis

XML Treatment for Stellaria longipedicellata

## ﻿Additional information

### Author ORCIDs

Wenqiao Wang https://orcid.org/0000-0002-8946-6995

Zhiwei Su https://orcid.org/0000-0003-2998-8617

Zhonghui Ma https://orcid.org/0000-0002-3898-3079

### Data availability

All of the data that support the findings of this study are available in the main text or Supplementary Information.

## Supplementary materials

Supplementary material 1 List of taxa sampled and GenBank accession numbers of sequences used in phylogenetic analyses of Alsineae Data type xlsx Explanation note This is a table including GenBank accession numbers of sequences used in phylogenetic analyses of Alsineae. https://binary.pensoft.net/file/1191142This dataset is made available under the Open Database License (http://opendatacommons.org/licenses/odbl/1.0/). The Open Database License (ODbL) is a license agreement intended to allow users to freely share, modify, and use this Dataset while maintaining this same freedom for others, provided that the original source and author(s) are credited.Wenqiao Wang, Zhonghui Ma
